# Use of TrueBeam developer mode for imaging QA

**DOI:** 10.1120/jacmp.v16i4.5363

**Published:** 2015-07-08

**Authors:** Gilmer Valdes, Olivier Morin, Yanisley Valenciaga, Niel Kirby, Jean Pouliot, Cynthia Chuang

**Affiliations:** ^1^ Department of Radiation Oncology University of California San Francisco CA; ^2^ Perelman Center for Advanced Medicine Radiation Oncology Department, University of Pennsylvania Philadelphia PA; ^3^ Biomedical Physics Interdepartmental Program David Geffen Medical School, University of California Los Angeles CA USA

**Keywords:** automated QA, TrueBeam developer mode, XML scripts, image‐guidance radiation therapy, support vector machine

## Abstract

The purpose of this study was to automate regular Imaging QA procedures to become more efficient and accurate. Daily and monthly imaging QA for SRS and SBRT protocols were fully automated on a Varian linac. A three‐step paradigm where the data are automatically acquired, processed, and analyzed was defined. XML scripts were written and used in developer mode in a TrueBeam linac to automatically acquire data. MATLAB R013B was used to develop an interface that could allow the data to be processed and analyzed. Hardware was developed that allowed the localization of several phantoms simultaneously on the couch. 14 KV CBCTs from the Emma phantom were obtained using a TrueBeam onboard imager as example of data acquisition and analysis. The images were acquired during two months. Artifacts were artificially introduced in the images during the reconstruction process using iTool reconstructor. Support vector machine algorithms to automatically identify each artifact were written using the Machine Learning MATLAB R2011 Toolbox. A daily imaging QA test could be performed by an experienced medical physicist in 14.3±2.4 min. The same test, if automated using our paradigm, could be performed in 4.2±0.7 min. In the same manner, a monthly imaging QA could be performed by a physicist in 70.7±8.0 min and, if fully automated, in 21.8±0.6 min. Additionally, quantitative data analysis could be automatically performed by Machine Learning Algorithms that could remove the subjectivity of data interpretation in the QA process. For instance, support vector machine algorithms could correctly identify beam hardening, rings and scatter artifacts. Traditional metrics, as well as metrics that describe texture, are needed for the classification. Modern linear accelerators are equipped with advanced 2D and 3D imaging capabilities that are used for patient alignment, substantially improving IGRT treatment accuracy. However, this extra complexity exponentially increases the number of QA tests needed. Using the new paradigm described above, not only the bare minimum — but also best practice — QA programs could be implemented with the same manpower.

PACS number: 87, 87.10.‐e

## I. INTRODUCTION

Modern linear accelerators are equipped with advanced 2D and 3D imaging capabilities that are used for patient alignment. Flat‐panel detectors have also increasingly been used for dosimetric applications. Task Group 179 and the AAPM Medical Physics Practice Guideline 2.a provide consensus recommendations for the quality assurance protocols of the imaging system of these linacs, especially those dedicated to image‐guided radiation therapy (IGRT).^1^, ^2^ For daily QA, tests to verify the imaging and radiation isocenter agreement, as well as the accuracy of the couch positioning and repositioning, are emphasized. For monthly QA, more comprehensive tests are proposed where not only agreement between the imaging and radiation isocenter, as well as the couch position and repositioning accuracy, are verified, but also extensive image quality tests and geometry calibration. These tests add between 3 to 5 hrs of extra monthly QA, according to TG‐179 and AAPM Medical Physics Practice Guideline 2.a.^1^, ^2^ Several efforts have been made to simplify the complexity of these tests. For instance, different commercial software and research articles have developed software that facilitates the processing of the data acquired in these QA procedures.^3^, ^4^ However, these approaches have been limited to the processing of the data, but the acquisition and the analysis of the same remain cumbersome. It is customary to use three to five different phantoms for the different tests with multiple setups. The interaction with the console for the delivery of the different tests is also far from optimal. Currently, each test needs to be delivered one at the time. Moreover, when the machines perform suboptimally, it is difficult to identify where the problem is and what course of action is required. Summarizing, an approach that combines hardware and software to facilitate the acquisition and processing of the data, as well as machine learning algorithms that facilitate the analysis of the data, is very much required. Therefore, in this work, we have defined a three‐step QA paradigm to facilitate these imaging QA tests on a Varian TrueBeam linac. These three steps are: 1) automatic data acquisition; 2) automatic data processing; and 3) automatic data analysis using machine learning algorithms.

It is expected that this paradigm will substantially reduce the time and errors due to human intervention involved in these QA procedures. Finally, it is worth mentioning that the paradigm proposed in this paper is somehow similar to the workflow employed for geometry calibration of the imagers using the Varian IsoCal phantom.^(5)^ However, in the present article, this approach will be extended to acquire all the tests required for daily and monthly QA at once and not only perform a geometry calibration.

## II. MATERIALS AND METHODS

In the present paper, daily and monthly imaging QA tests for the imaging systems of a TrueBeam linac (Varian Inc., Palo Alto, CA) were automated. These tests were performed three times by an experienced medical physicists and the time recorded. These times were in all cases comparable to those reported by the AAPM Medical Physics Practice Guideline 2.a for similar tests.^(1)^ The same tests were also fully automated, repeated three times, and measured for comparison. For the automatic tests, the interaction with the console of the TrueBeam was completely done through XML scripting according to TrueBeam Developer Mode 2.0 (Varian Inc.). All the XML scripts described in the present paper could be found at the repository https://github.com/valdesg/Automatic‐True‐Beam‐QA. The XML scripts have been published individually so that interested readers could combine them according to their specific phantoms, mounts, and QA protocols, as described below. Additionally, in some cases, MATLAB functions (Math Works, Natick, MA) have been included that allow the manipulation of the XML files. On the other hand, a summary of these tests and their implementation is given. The daily QA consisted of two tests as described below.

**Figure 1:**
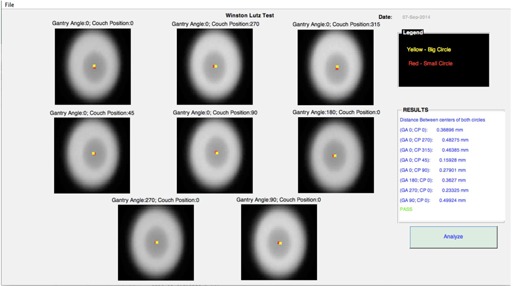
MATLAB GUI for the Winston‐Lutz test. Images of the Winston‐Lutz test kit are shown.

1 Test 1: Imaging and treatment coordinate coincidence. After the initial geometric calibration has been performed using procedures similar to that described by Bissonnette et al.,^(2)^ a daily geometric accuracy QA consists of stability and consistency tests is performed. The MIMI phantom (Standard Imaging Inc., Middleton WI), which has embedded BBs, was used to take portal images and CBCT, and the positions of the embedded BBs on these daily images as compared to the base line were used to verify the imager's consistency.
2 Test 2: Couch position and repositioning. The same phantom used in the imaging and treatment coordinate coincide is used in this test. The same is placed at the physical isocenter. A specific displacement of the couch from isocenter using a random number generator is applied (the movement in all axis is within 2 cm to be in agreement with TG‐179^(2)^). Then, a localization CBCT image dataset to assess the couch motion required to align the phantom with a reference CT dataset is acquired. The registration of the images for the position and repositioning tests are done automatically. The couch is then shifted and a new verification dataset acquired to verify that the couch shift has corrected the positioning difference. This value should be near 0±2 mm for regular IGRT, and 0±1 mm for SBRT treatment, according to AAPM Medical Physics Practice Guideline 2.a, TG‐179 and TG‐142.^1^, ^2^, ^6^ When these tests were performed automatically, a three‐step paradigm, as mentioned above, was used. Two scripts, one for each test, were written. Both scripts were then combined to produce one XML script that could allow the acquisition of the data in both tests simultaneously. No software was written for the processing of the data acquired in the Daily QA. The time for processing in this case was not evaluated, as the developer mode does not allow integration with the 2D/2D or 3D/3D match algorithm. However, this process was measured offline and added to the acquisition time to estimate the total time of the daily QA. Additionally, the Winston‐Lutz test was also implemented and its duration added to the daily QA time for those days where this test is required, as it is standard in our clinic to perform this test on those days where cones are used for SRS treatment. The Winston‐Lutz test was performed as described by Lutz et al.(7) using the Winston‐Lutz test kit from BrainLAB (BrainLAB, Feldkirchen, Germany). The processing and analysis of the data was performed by an in‐house MATLAB GUI (MATLAB R2013B) developed to automatically find the center of radiation defined by the cone and the center of the ball bearing (Fig. 1). The center of the bigger circle was found by finding the geometric center of the pixels with levels between 2500 and 2700, while the center of the smaller circle was found by finding the geometric center of the pixels with level between 1700 and 2200. Both centers are always shown in the images for visual inspections. Additionally, the positions of the centers were validated against the position of the centers that are obtained when the imaging analysis tool of the Varian TrueBeam developer console is used. In both cases, there were an agreement of 0.1 mm between both methods. This software could also be found at the mentioned repository. Additionally, a video of this test being performed automatically could be seen at http://goo.gl/Hvygni.

On the other hand, the monthly imaging QA was performed according to AAPM Medical Physics Practice Guideline 2.a, TG‐179, and TG‐142.^1^, ^3^, ^6^ The procedure consisted of four tests, as described below.

### A. Image quality test

**Figure 2:**
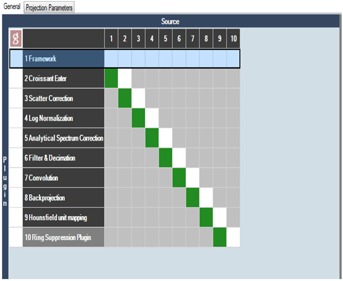
Default head reconstruction chain in iTools reconstruction (Varian Inc.). Data flow from the first to the final plug‐in. If plug‐ins are suppressed, artifacts are created in the images.

**Figure 3:**
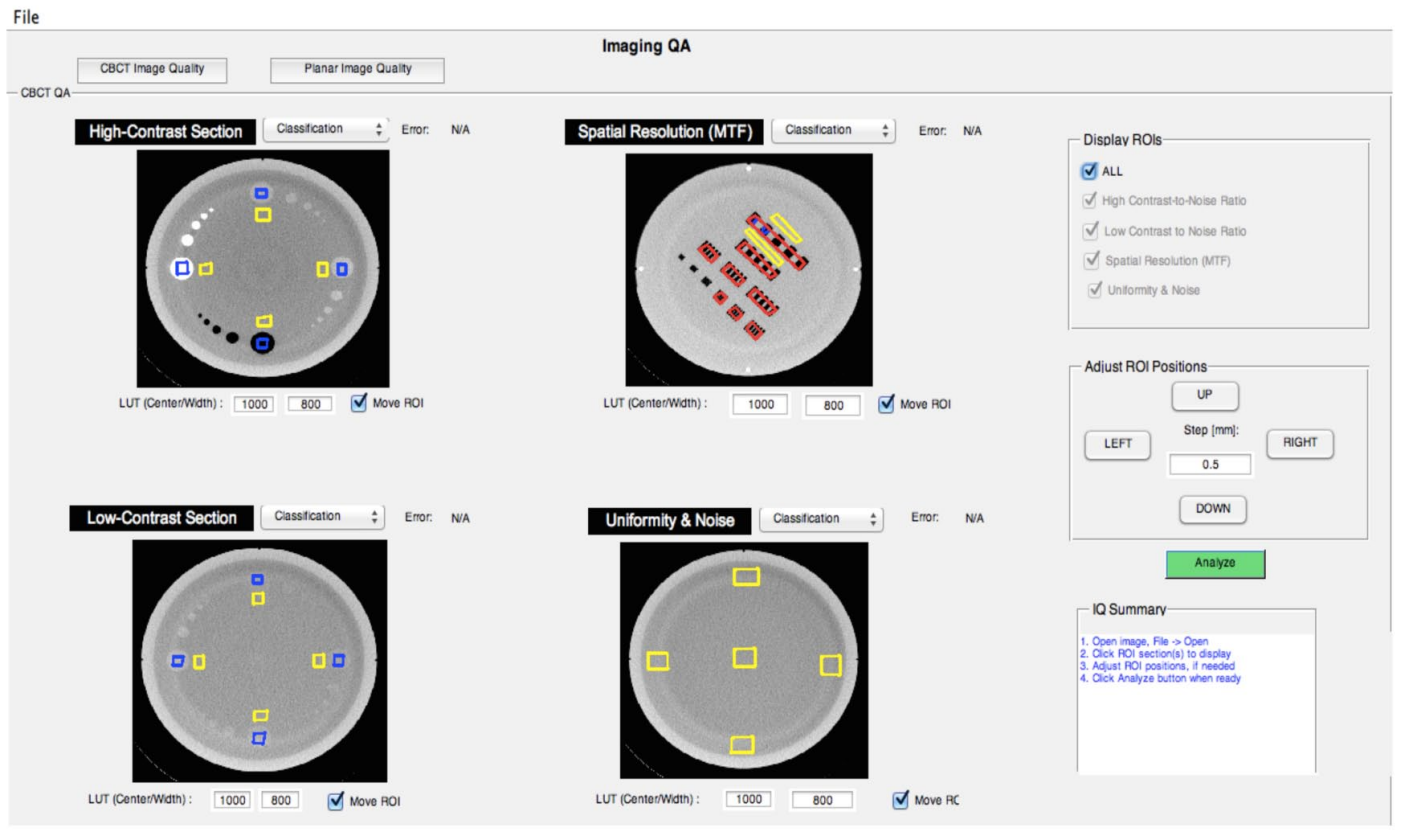
MATLAB GUI for image quality analysis. Images have been taken from different sections of the Emma phantom.

A CBCT is acquired using the EMMA phantom (Siemens Healthcare, Erlangen, Germany). A detailed description of this phantom was given by Sumida et al.^(8)^ Briefly, this phantom consists of four sections: high contrast, low contrast, spatial resolution and uniformity, and noise. In order to establish baselines and develop algorithms to automatically identify artifacts in the CBCT images, 14 KV CBCT of the EMMA phantom were acquired during two months using a TrueBeam. The technique used to acquire the CBCT was a full head scan consisting of full acquisition mode, 40 mA and 10s pulses, 25 cm field of view, and 100 KV. The projections were reconstructed and the CTs obtained using the default head reconstruction chain from iTool reconstruction 2.0 (Varian Inc.), shown in Fig. 2. (iTool reconstruction software was provided by Varian Inc. through our research agreement, and it is not part of the Developers Mode.) To obtain clinically relevant artifacts, the reconstruction chain from iTool was modified. Artifacts were obtained by eliminating the corresponding plug‐in from the reconstruction chain (beam hardening, rings, scatter, and crescent artifact). Additionally, images with multiple artifacts present at the same time were also created and included in the dataset to evaluate how the presence of multiple artifacts in the images would affect the identification of different artifacts. When the quality of a CBCT was evaluated, different features would be calculated from each of these sections to compare to baseline using an in‐house MATLAB GUI. In the case of the high‐contrast resolution and low‐contrast resolution sections, contrast‐to‐noise ratio (CNR) would be calculated according to Gayou and Miften^(9)^ for the four 2 cm rods respectively. The spatial resolution section would be used to calculate the modulation transfer function at 50% and 10% (MTF50 and MTF10), according to Doeger and Morin^(10)^ Finally, uniformity and noise would be calculated from the section with the same name according to Sumida et al.^(8)^ Besides these magnitudes, each of these sections was further characterized with 99 texture features: 5 statistics features (mean, standard deviation, entropy, skewness, and kurtosiss); 76 features from four grey co‐occurrence matrixes corresponding to 8 pixel leaves and 0°, 45°, 90°, and 315° direction with neighbor pixels; and 18 scale features (first order, gradient, and second order features) corresponding to three scales (1, 2, 4) as described in different papers.^11^, ^12^, ^13^ In all texture calculations, the whole section would be used as the ROI to calculate the features. A vector combining the texture and the more classical features described above for each of the section of the Emma phantom was used to describe each phantom section for each KV CBCT image. A classifier was then written, using one classification vs. all support vector machine (SVM) algorithms. This algorithm uses the vector of features mentioned above to classify the images according to the type of artifact present. In our case, a linear kernel was used in the SVM algorithm, as the data were linearly separable in the initial feature space. A sequential forward selection was performed to find the features that best describe each section and artifact using, as the objective, the minimization of a ten‐fold classification cross‐validation error. A separate classifier for each section of the Emma phantom was obtained in all cases. Additionally, to test the quality of the planar KV and MV images, the QCkV‐1 and QC‐3 phantoms (Standard Imaging, Inc.) were used. These phantoms are described by Kim et al.^(14)^ Similar magnitudes, as described by the CBCT, are calculated for both the MV and the KV phantoms. The ROIs in these phantoms are also automatically detected using the geometry characteristics of the phantoms and the known distances of each phantom respective to each other. When the physicist performed these tests, each of the phantoms had to be localized individually with multiple entrances to the treatment room after each test. In all cases, these features were calculated using our in‐house MATLAB GUI (Fig. 3). Tests of imaging scaling were not included in this set of tests, as our initial tests focused mostly on new imaging data compared with baseline data in terms of signal to noise, contrast, and resolution.^(1)^ However, we are currently working on the inclusion of imaging scaling as part of the updated monthly procedures, and we do not think that it will present major complexity.

### B. Image registration and correction test

A phantom is placed on the couch with known position in respect to machine isocenter. The couch positions are automatically displaced to five controlled positions and a KV OBI, MV EPID, and kV‐CBCT are obtained for each couch position. The shifts reported by the 2D/2D and 3D/3D image registration software after the analysis is compared with the known shifts.

### C. Geometry calibration, EPID position, and reproducibility tests

For the case of geometry calibration and EPID position and reproducibility tests, the IsoCal phantom was used and the calibration performed, as reported by Gao et al.^(5)^

**Figure 4:**
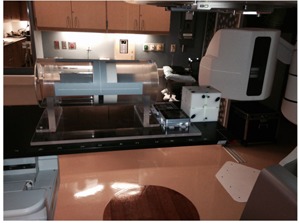
In‐house mount that allows the localization of all phantoms used in the monthly QA in the couch at the same time. In the figure, from left to right we find the Emma, QCkV‐1, QC‐3, and MIMI phantoms.

When these tests were performed automatically, a mount developed in our lab that allows the localization of each phantom with respect to each other on the couch was used (Fig. 4). Then, two XML scripts that acquired several tests consecutively were written.

The first XML script will only acquire the image quality and the image registration and correction tests, as described above, and the other will acquire, in addition, the data needed to perform the geometry calibration and the EPID position and reproducibility tests as if the phantoms that we were using were suited for this task. This test will simulate the monthly imaging QA if a universal phantom is built. The processing of the data for the tests B, C and D was not evaluated automatically, as the developer mode does not allow integration with the 2D/2D and 3D/3D matching algorithm. However, as in the case of the daily QA, these processes were measured offline and added to the acquisition time to estimate the total time of the monthly QA after being fully automated. The image quality data were processed using our in‐house MATLAB GUI, as described above.

Finally, it is important to notice these XMLs included in this paper localize the different phantoms taking into account the specific geometric characteristics of the phantoms and the mount that was used. However, different clinics could use different phantoms and mounts by simply changing the couch coordinates on the XML files without affecting the overall code. Possible collisions should always be ruled out.

## III. RESULTS

### A. Daily QA without Winston‐Lutz test

A daily QA was performed by the physicist, as described in the Materials and Methods section. In this case, the same phantom could be used to perform both tests required for the daily QA, but the physicists still needs to enter the treatment room to reposition the couch after the first test. The time that it takes for an experienced physicists to perform this test is reported in Table 1. On the other hand, both tests were automated and the time it takes to acquire the data using an XML script recorded. In this case, the physicist only needs to upload the XML script, move the axes to plan, and deliver the beam, which requires less intervention from his side. The time it takes the acquisition of the data was also recorded and the processing time, as explained in the Materials and Methods section, added to Table 1. A total of 10.1 min is saved when the daily QA is fully automated. Additionally, the workflow is simplified, as explained in the Materials and Methods section.

**Table 1:** Imaging QA time (mins), physcist vs. full automation

*QA*	*Physicists*	*Full Automation*
Daily QA	14.3±2.4	4.2±0.7
Winston‐Lutz Test	29.1±6.2	3.1±0.9
Imaging monthly QA without geometry calibration and EPID position and reproducibility	58.7±6.6	19.3±1.0
Imaging monthly QA	70.7±8.0	21.8±0.6

### B. Winston‐Lutz test

For SRS cases the Winston‐Lutz test is recommended.^(7)^ This test was performed by a physicist, as described in the Materials and Methods section. Additionally, the same was fully automated, as described below, using our three‐step paradigm.

1 Step 1: automatic data acquisition. Using XML scripts, eight planar MV images at eight different combinations of gantry and couch positions were automatically acquired.
2 Step 2: automatic data processing. An in‐house software (MATLAB GUI) that reads the eight images and finds the distance between the centers of the ball bearings and the radiation cones for each image was developed (Fig. 1).
3 Step 3: automatic analysis and report generation. Decision regarding the success of the test presented and complete PDF document generated and uploaded to EMR.

The comparison of the time it takes an experienced medical physicist to perform this test vs. the time it takes if the test is fully automated is shown in Table 1. Additionally, the workflow is greatly simplified with respect to the current method used in our clinic, as explained in the Materials and Methods section.

### C. Monthly QA without geometry calibration and EPID position and reproducibility

A monthly QA was performed by the physicist, as described in the Materials and Methods section. The same test was also performed automatically. In this case, all phantoms that are required to perform the test were localized on the couch at the same time using a mount that we designed (Fig. 4) All the data were acquired at once using an XML script. The image quality data were processed with our in‐house software (Fig. 3). Using machine learning algorithms, the images were classified and artifacts automatically detected, as described in the Results section E below. The data from the image registration and correction were acquired using the same XML script, but not processed. However, the time was estimated as described in the Materials and Methods section. The comparison of the time it takes an experienced medical physicist to perform a monthly QA without geometry calibration and EPID position and reproducibility vs. the same QA fully automated is shown in Table 1

### D. Monthly QA with geometry calibration and EPID position and reproducibility

As was described above, using the IsoCal phantom and the software that comes with it, the geometry calibration and EPID position reproducibility has been automated by the integration of hardware, beam delivery, and software at the console; a very similar way as the one we are proposing here for all other QA tests. However, in order to simplified this process, one could imagine a one‐stop phantom that could allow all the monthly imaging QA tests to be acquired at once and not only the geometry calibration. In fact, the Emma phantom already has 12 ball bearings that could be used with the right software to geometrically calibrate the KV CBCT system and the EPID positions of TrueBeam. As in the other cases, physicist performance vs. fully automated performance is compared in Table 1

### E. Automatic artifact detection and data analysis using machine learning

**Figure 5:**
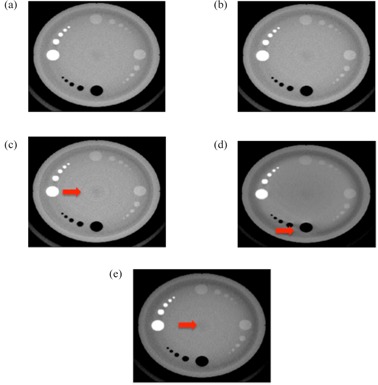
KV CBCT artifacts produced after the plug‐in from the iTool reconstruction chain are suppressed. All images correspond to the high‐contrast section from the Emma phantom. Red arrows indicate the artifacts in the images: (a) regular image; (b) crescent artifact; (c) rings; (d) scatter; (e) beam hardening.

**Figure 6:**
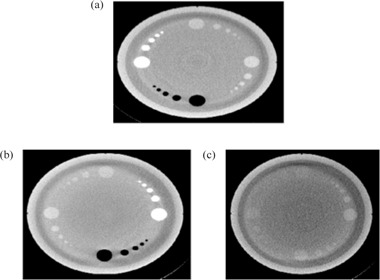
KV CBCT images with multiple artifacts. The presence of different artifacts in an image did not prevent the algorithm from identifying each individually. Beam hardening and rings (a); scatter and beam hardening (b); scatter and rings (c). Images correspond to the Emma phantom.

Figure 5 shows images of the different artifacts that were introduced during the reconstruction process to simulate artifacts that are usually found in clinical images. Additionally, a regular image is also included for comparison. As it can be seen, the images that were supposed to have crescent artifacts cannot be differentiated from regular images. In the same way, the SVM machine algorithms could not separate crescent artifacts from regular images. On the other hand, scatter, rings, and beam hardening artifacts using different sections of the Emma phantom could all be correctly identified by the SVM algorithms, even when more than one artifact was present in the image at the same time (Fig. 6). The ten‐fold, cross‐validation errors obtained for the identification of the different artifacts and regular images per section of the phantom are shown in Table 2. Beam hardening artifacts could be identified using any of the sections from the Emma phantom, while rings and scatter could be corrected classified using the low‐contrast section and the spatial resolution or high‐contrast section, respectively. For crescent artifacts, a high classification error is obtained when regular images are part of the dataset. However, almost perfect classification is obtained (cross‐validation error less than 0.01) when the regular images are grouped with the crescent artifacts, which indicates that in our dataset, these categories are really the same. Additionally, similar result is obtained if, instead of a linear kernel, a radial basis function kernel is used, which indicates that the reason why the regular and crescent artifacts are not separable by our algorithm is not because a linear classifier was used. This is also apparent in Fig. 5. On the other hand, the features selected by the sequential forward selection to identify the other type of artifacts are shown in Table 3. Interesting enough, several texture features that are usually not calculated to describe KV CBCT images were selected by the algorithms to identify different artifacts.

**Table 2:** Ten‐fold, cross‐validation errors of the classification of different artifacts and regular images by the classifiers obtained by a SVM algorithm. For each section of the phantom, a different classifier is used

	*Uniformity and Noise*	*Spatial Resolution*	*High‐Contrast Section*	*Low‐Contrast Section*
Scatter	0.03	0	0	0.02
Beam Hardenig	0	0	0	0
Rings	0.2	0.06	0.03	0
Crescent Artifacts	0.45	0.36	0.39	0.38
Regular	0.1	0.14	0.23	0.28
Regular and Crescent Artifact	0.18	0.03	0.01	0.18

**Table 3:** Features per section selected by the sequential forward selection algorithm to classify the different artifacts

	*Uniformity and Noise*	*Spatial Resolution*	*High‐Contrast Section*	*Low‐Contrast Section*
Scatter	dissimilarity	skewness	CNR 3 mm, inverse different moment normalized	kurtosiss
Beam Hardening	uniformity, mean	mean	mean	skewness
Rings	scale features	scale features	CNR 3 mm, information measurement correlation 1 and 2, inverse difference and scale features	CNR 3 mm, scale features
Crescent Artifacts	entropy, difference entropy	inverse different moment normalized	correlation, homogeneity	entropy from GLCM
Regular	noise, entropy	mean, scale features	correlation	contrast
Regular and Crescent Artifact	noise	Homogeneity, Scale features	mean, STDV, homogeneity, scale features	CNR 9 mm structure

## IV. DISCUSSION

Today's imaging QA procedures for on‐board imaging systems are far from optimal. Several different phantoms with different software packages have to be used, which makes the process time‐consuming, complex, and physically demanding. As it has been shown in this paper, these processes could be simplified with the right combination of software and hardware. Our three‐step QA paradigm — 1) automatic data acquisition, 2) automatic data processing, and 3) automatic analysis and reporting — could not only substantially reduce the time that these procedures take (as seen in Table 1), but also reduce the complexity of the process, which brings several advantages. For instance, in Table 1, the time that it takes an experienced physics to perform the tests has been computed, assuming there are not errors in the process. However, due to the complexity of the procedures, the physicists could make several mistakes. A planar MV image could be taken with the collimator and leafs closed. This error was actually made while we were timing the physicist performing the QA. However, we did not take this into consideration when we estimated the time. These types of errors are avoided in the automatic acquisition because the correct positions for all axes could be specified on the XML file. Other random errors could also be eliminated which could bring higher standardization to the QA process. Additionally, because the workflows of the tests are substantially simplified, they could be performed faster and would be less prone to errors, and different personnel, not only qualified medical physicists, might be able to perform the tests. This is already the case for the daily QA where in most clinics the therapists perform the tasks. This could help to address the issue of scarce resources for centers where resources, especially qualified medical physicists' time, is limited. Moreover, the simplification of both daily and monthly imaging QA opens the possibility of performing the monthly imaging QA once a month during the time of daily QA. On the other hand, with the use of machine learning, previous qualitatively processes could be replaced by quantitative approaches. In this paper, modifying the reconstruction chain in the iTool 2.0 reconstructor from Varian produced several artifacts. The data were then used as a proof of principle to show that image quality artifacts could be automatically detected, eliminating the subjectivity involves when the images are qualitatively evaluated. As it is shown in Table 2, the presence of one type artifact in an image does not prevent the correct classification of another artifact. This indicates that, even in the case of multiple problems happening with the imaging device, all of them could be identified. However, the crescent artifact and the regular images could not be separated and correctly classified. Regular images could be correctly classified with probability of 99%, if the crescent artifacts were not present or labeled as regular images. As it is shown in Fig. 5, there was also no apparent difference between the regular images and the crescent artifacts. This artifact is caused by the wiggling of the bowtie filter during gantry rotation to acquire all the projections needed for the reconstruction of the CBCT. The crescent artifact is then corrected using the geometry calibration of the linac. The fact that no realistic crescent artifact was obtained after the geometric calibration was suppressed from the reconstruction chains indicates that there is little sagging and wiggling of our linac. However, there is no reason to believe that, in a dataset where this artifact is present, a classifier that identifies this artifact could not be obtained using the procedure described in this paper. On the other hand, it is also very important to highlight that, for the cases of the artifacts that were correctly classified by the algorithm, some of the features selected for their classification are not currently calculated in clinical settings when the image quality of the CBCTs are evaluated. Since they are the best features to minimize classification errors, the question of whether they should be calculated or not is very important. This work strongly supports their inclusion as part of the regular metrics in any image QA evaluation process.

Finally, there are several reasons to have an automatic artifact identification process in place. First, the subjectivity of the task as performed today is eliminated. Second, we hypothesize that, because the noise in all metrics of our images was low, we might be able to identify problems before they manifest clinically. For this endeavor, a time tracking of IQ data in the clinic should be performed. This hypothesis will be tested in future experiments.

Additionally, it would also be important to establish whether the features obtained using the data for one linac could be used for other linacs, or if the algorithms should be trained for each linac specifically. In the near future, a multi‐institutional study where artifact data is collected and the algorithms trained to classify them should be performed. Additionally, even though this paper has focused on image quality, automatic QA and machine learning could play a similar role in other aspects of quality assurance in medical physics. As it was shown in this paper, not only full automatization of the QA process is possible, but also a revaluation of the metrics used to describe the processes could be established. It is the opinion of these authors that the time when one phantom is placed in the table that has both imaging sections and ion chambers and a full monthly QA is acquired in 1 hr after one button is pressed is on the near horizon. For this vision to become reality, a vendor should develop such a phantom targetted to the monthly QA and newer generations of linacs should include developer mode.

## V. CONCLUSIONS

With the development of hardware and software, the QA procedures could be simplified. However, the involvement of the linac vendors, and not only of third‐party companies, is needed for a better integration and solution. Additionally, this article serves as a proof of principle that image artifacts could be automatically detected and the troubleshooting process facilitated using machine learning algorithms on a Varian TrueBeam linac. In the same manner, new metrics that are not normally calculated in IQ processes should be incorporated.

## ACKNOWLEDGMENTS

This work was supported by a research grant from Varian Medical Services, Palo Alto, California.

## References

[1] Fontenot JD , Alkhatib H , Garrett JA , et al. AAPM Medical Physics Practice Guideline 2.a: Commissioning and quality assurance of X‐ray‐based image‐guided radiotherapy systems. J Appl Clin Med Phy. 2014;15(1):4528. 10.1120/jacmp.v15i1.4528 PMC5711227 24423852

[2] Bissonnette JP , Balter PA , Dong L , et al. Quality assurance for image‐guided radiation therapy utilizing CT‐based technologies: a report of the AAPM TG‐179. Med Phys. 2012;39(4):1946–63. 22482616 10.1118/1.3690466

[3] Gopal A and Samant SS . Use of a line‐pair resolution phantom for comprehensive quality assurance of electronic portal imaging devices based on fundamental imaging metrics. Med Phys. 2009;36(6):2006–15. 19610289 10.1118/1.3099559

[4] Das IJ , Cao M , Cheng CW , et al. A quality assurance phantom for electronic portal imaging devices. J Appl Clin Med Phys. 2011;12(2):3350. 21587179 10.1120/jacmp.v12i2.3350 PMC5718680

[5] Gao WD and Balter P . Evaluation of IsoCal imaging isocenter calibration system for Varian OBI machines [abstract]. Med Phys. 2012;39(6):3604.

[6] Klein EE , Hanley J , Bayouth J , et al. Task Group 142 report: quality assurance of medical accelerators. Med Phys. 2009;36(9):4197–212. 19810494 10.1118/1.3190392

[7] Lutz W , Winston KR , Maleki N . A system for stereotactic radiosurgery with a linear accelerator. Int J Radiat Oncol Biol Phys. 1988;14(2):373–81. 3276655 10.1016/0360-3016(88)90446-4

[8] Sumida I , Yamaguchi H , Kizaki H , et al. Evaluation of imaging performance of megavoltage cone‐beam CT over an extended period. J Radiat Res. 2014;55(1):191–99. 23979076 10.1093/jrr/rrt100 PMC3885132

[9] Gayou O and Miften M . Commissioning and clinical implementation of a mega‐voltage cone beam CT system for treatment localization. Med Phys. 2007;34(8):3183–92. 17879781 10.1118/1.2752374

[10] Droege RT and Morin RL . A practical method to measure the MTF of CT scanners. Med Phys. 1982;9(5):758–60. 7155079 10.1118/1.595124

[11] Tourassi GD . Journey toward computer‐aided diagnosis: role of image texture analysis. Radiology. 1999;213(2):317–20. 10551208 10.1148/radiology.213.2.r99nv49317

[12] Clausi DA . An analysis of co‐occurrence texture statistics as a function of grey level quantization. Can J. Remote Sensing. 2002;28(1):45–62.

[13] Soh LK and Tsatsoulis C . Texture analysis of SAR sea ice imagery using gray level co‐occurrence matrices. IEEE Trans Geosci Remote Sensing. 1999;37(2).

[14] Kim KF , Lazos D , Harrison L . An intercomparison of imaging performance of two linac‐mounted imaging systems used in radiation therapy: TrueBeam and Trilogy [abstract]. Med Phys. 2012;39(2):3656. 10.1118/1.4734854 28517576

